# Association of Radial Artery Access with Reduced Incidence of Acute Kidney Injury

**DOI:** 10.1155/2023/1117379

**Published:** 2023-01-18

**Authors:** Patrick S. Kietrsunthorn, Tonja M. Locklear, Clifford E. Fonner, Chalak O. Berzingi, Jason R. Foerst, Mohd A. Mirza, David C. Sane, Eric Williams, Robert A. Shor, Gregory J. Dehmer

**Affiliations:** ^1^Cardiology Division, Carilion Clinic, Virginia Tech Carilion School of Medicine, Roanoke, VA, USA; ^2^Health Analytics Research, Virginia Tech Carilion School of Medicine, Carilion Clinic, Roanoke, VA, USA; ^3^Virginia Cardiac Services Quality Initiative, South Riding, VA, USA; ^4^Virginia Heart, Reston, VA, USA

## Abstract

**Objectives:**

To determine if radial artery (RA) access compared with femoral artery (FA) access for percutaneous coronary intervention (PCI) is associated with a lower incidence of acute kidney injury (AKI).

**Background:**

AKI results in substantial morbidity and cost following PCI. Prior studies comparing the occurrence of AKI associated with radial artery (RA) versus femoral artery (FA) access have mixed results.

**Methods:**

Using a large state-wide database, 14,077 patients (8,539 with RA and 5,538 patents with FA access) were retrospectively compared to assess the occurrence of AKI following PCI. To reduce selection bias and balance clinical data across the two groups, a novel machine learning method called a Generalized Boosted Model was conducted on the arterial access site generating a weighted propensity score for each variable. A logistic regression analysis was then performed on the occurrence of AKI following PCI using the weighted propensity scores from the Generalized Boosted Model.

**Results:**

As shown in other studies, multiple variables were associated with an increase in AKI after PCI. Only RA access (OR 0.82; 95% CI 0.74–0.91) and male gender (OR 0.80; 95% CI 0.72–0.89) were associated with a lower occurrence of AKI. Based on the calculated Mehran scores, patients were stratified into groups with an increasing risk of AKI. RA access was consistently found to have a lower risk of AKI compared with FA access across these groups of increasing risk.

**Conclusions:**

Compared with FA access, RA access is associated with an 18% lower rate of AKI following PCI. This effect was observed among different levels of risk for developing AKI. Although developed from a retrospective analysis, this study supports the use of RA access when technically possible in a diverse group of patients.

## 1. Introduction

Acute kidney injury (AKI) affects morbidity and mortality in patients with acute coronary syndromes and those who undergo percutaneous coronary intervention (PCI) [1–3]. The development of AKI in such patients can lead to chronic or end-stage renal failure [4]. Besides being a strong predictor of in-hospital and 1-year mortality in this patient population, AKI can increase costs due to an increased length of stay and hospital readmission [3, 5].

There are several definitions of AKI following the administration of radiographic contrast agents. The National Cardiovascular Data Registry (NCDR) has adopted the Acute Kidney Injury Network criteria which defines AKI by any of the following: (1) increase in serum creatinine of ≥0.3 mg/dL from the baseline, (2) increase in serum creatinine of 50% or more from the baseline, or (3) new requirement for dialysis [6]. Using this definition, about 7% of patients develop AKI and 0.3% of patients require new dialysis after PCI [7]. There are several predictors for the development of AKI including a reduced baseline estimated glomerular filtration rate (eGFR), cardiogenic shock, and the amount of contrast administered [5, 7, 8].

Some prior studies found that radial artery (RA) access compared with femoral artery (FA) access is associated with a lower occurrence of AKI following PCI [9–11]. However, this has not been a consistent finding among studies and varies depending on the definition of AKI used and the population studied [12]. Mixed results are also present when the population studied was restricted STEMI patients undergoing PCI with two studies showing no difference in the occurrence of AKI between RA versus FA access, but one study showing an advantage of RA access in STEMI patients [9, 13, 14]. Despite several studies investigating the relationship between the access site and AKI, there is no consensus. This may be due to the multifactorial causes of AKI after PCI and the challenges of controlling for confounding factors (such as baseline renal function, bleeding, and shock). A large, randomized trial of RA versus FA access specific for a reduction of AKI would be ideal but is unlikely as the accepted advantages of RA access on mortality, vascular complications, and bleeding would hamper recruitment [15–17]. Using a large database, the purpose of this retrospective study was to examine the effect of the access site on the incidence of AKI following PCI.

## 2. Materials and Methods

### 2.1. Study Population

The study population was derived from a state-wide collaborative group of 18 interventional cardiology centers within Virginia. This effort, known as the Virginia Cardiac Services Quality Initiative (VCSQI), aggregates deidentified data collected from the NCDR CathPCI Registry to assist facilities in benchmarking and quality improvement efforts. Collectively, VCSQI centers perform approximately 75% of the PCI procedures in the Commonwealth. Member institutions and the VCSQI maintain business associate agreements with the database vendor (ARUMUS Corporation, Foster City, CA). Consent for the use of these deidentified data is covered under an agreement between the NCDR and VCSQI; thus, the local institutional review was not required.

**Figure 1:**
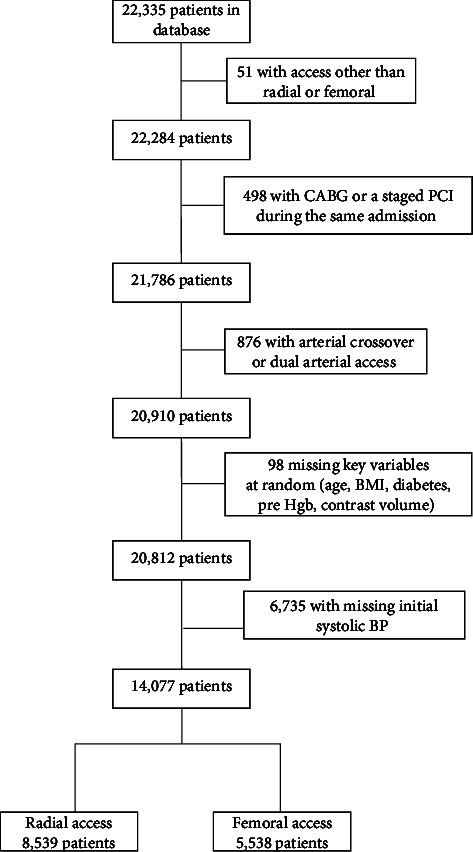
Flowchart of excluded patients. Legend: patient inclusion/exclusions in the consolidated standards of reporting trials (CONSORT) format. Abbreviations: BMI = body mass index; BP = blood pressure; CABG = coronary artery bypass graft; Hgb = hemoglobin; PCI = percutaneous coronary intervention.

Beginning January 1, 2017, and through December 31, 2020, 32,740 records from patients undergoing PCI were aggregated in the VCSQI database. After excluding records missing a creatinine value before or after the PCI, 22,335 records remained. Additional patient records were excluded from the final cohort as outlined in Figure 1. The largest number of records excluded (*n* = 6,735) were missing the first blood pressure reading recorded in the procedure room. This occurred because data collection spanned the change from Version 4 to Version 5 of the CathPCI Registry which occurred in April 2018. The first blood pressure reading was not collected in Version 4, and this accounted for 100% of the 6,735 missing values. This same variable was collected in 99% of the records from Version 5. The systolic blood pressure reading was entered as a binary response indicating the presence (<90 mmHg systolic) or absence of hypotension (≥90 mmHg) at the start of the procedure. The final cohort for analysis consisted of 14,077 patients: 8,539 with RA access and 5,538 with FA access. The presence of AKI following PCI was determined using the definition specified by the NCDR as an absolute increase of ≥0.3 mg/dL or a relative increase of 50% in serum creatinine or a new requirement for dialysis following PCI [6, 18]. Definitions of the other variables were established by the NCDR [19]. Follow-up creatinine measurement after PCI was typically within the first 5 days but was not standardized among the institutions. The type, amount, and duration of hydration following PCI were determined by individual operators and were not standardized.

The risk of AKI in patients was estimated using an established scoring system (Mehran scores) [20]. Four groups, each with a progressively increasing risk of AKI, were defined based on a point score derived from the selected clinical variables. The occurrence of AKI in patients with RA or FA access was compared in each risk group to determine if there was a benefit of RA access on the incidence of AKI at different degrees of risk.

### 2.2. Statistical Analysis

As expected when using a large, retrospective database, some data fields were incomplete as noted in Figure 1. Moreover, some data fields containing overlapping information were combined for data entry and, in some cases, transformed into a binary (yes/no) response. An explanation of the processes used for data translation is provided as Supplementary Table S1. To reduce selection bias and balance clinical data across the different access groups, a machine learning method called a Generalized Boosted Model (GBM) was conducted on the arterial access site by first choosing variables that would have been available before the selection of the access site [21–23]. Other variables were added one at a time until the model obtained its best fit based on the standardized mean differences between the RA and FA weighted variables reaching <10% (Supplementary Table S2).

A logistic regression analysis was performed on the occurrence of AKI following PCI using the weighted propensity scores from the GBM. All fields except for eGFR were included in the logistic regression because eGFR is determined from a combination of variables already included in the analysis (serum creatinine, age, gender, and race). In addition, the Mehran risk scores were not included in the GBM or logistic regression due to the amalgamated nature of that score value from variables already included. Comparison of the rates of AKI in the groups defined by the Mehran risk scores was performed using Fisher's Exact Test. All statistical analyses were performed using R Studio (Version 1.2.1335) with a significance level of 5% [24].

## 3. Results

### 3.1. Variables Associated with AKI

**Figure 2:**
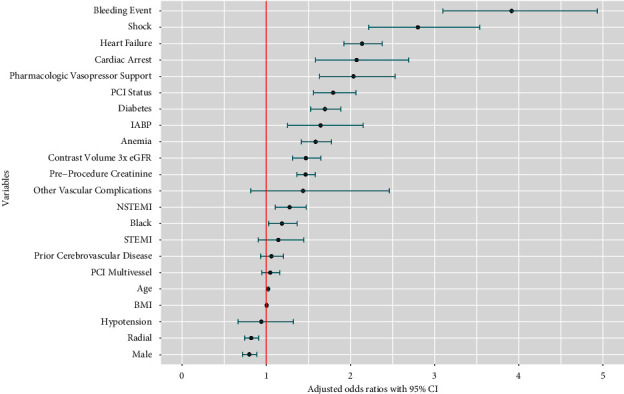
Odds ratio from logistic regression on AKI after PCI with propensity score weights. Abbreviations: AKI = acute kidney injury; BMI = body mass index; CI = 95% confidence interval; eGFR = estimated glomerular filtration rate; IABP = intra-aortic balloon pump; NSTEMI = non-ST-segment elevation myocardial infarction; PCI = percutaneous coronary intervention; STEMI = ST-segment elevation myocardial infarction.

**Figure 3:**
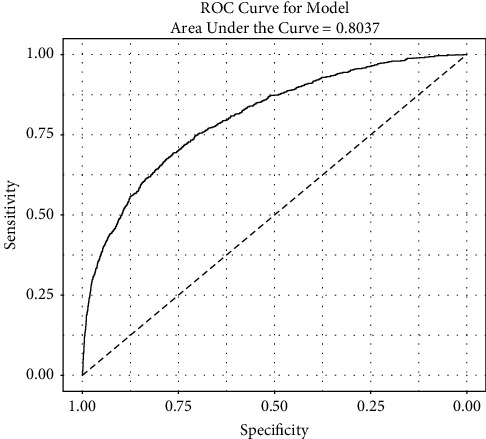
The receiver operator characteristic curve for the logistic model. The C-statistic is 0.8037. Abbreviations: ROC = receiver operator characteristic.

Results of the logistic regression model are shown in Table 1 and Figure 2. The C-statistic was 0.804 for the logistic regression model (Figure 3). Multiple variables were associated with an increased occurrence of AKI following PCI. The most impactful were a postprocedure bleeding event (odds ratio (OR): 3.94; 95% confidence interval (CI): 3.13–4.97), presence of shock (OR: 2.82; 95% CI: 2.24–3.57), presence of heart failure (OR: 2.14; 95% CI: 1.93–2.38), presence of cardiac arrest (OR: 2.08; 95% CI: 1.59–2.70), use of pharmacologic vasopressor support (OR: 2.03; 95% CI: 1.63–2.53), higher acuity of PCI status (1-elective, 2-urgent, 3-emergency, 4-salvage) (OR: 1.78; 95% CI: 1.55–2.05), presence of diabetes (OR: 1.69; 95% CI: 1.52–1.88), use of an intra-aortic balloon pump (OR: 1.63; 95% CI: 1.24–2.14), and presence of anemia (OR: 1.58; 95% CI: 1.41–1.77) (Table 1). Other variables associated with an increase in AKI were preprocedure creatinine and contrast volume administered exceeding 3 times the eGFR, NSTEMI, Black race, and age (Table 1). The radial access site (OR: 0.82; 95% CI: 0.74–0.91) and male gender (OR: 0.80; 95% CI: 0.72–0.89) were the only factors associated with a lower occurrence of AKI. The need for dialysis following PCI was also higher with FA compared with RA access (0.9% vs. 0.4% and *p* < 0.001). As a measure of a facility's experience with radial access, we separated the 18 facilities into 2 groups: those with >50% of cases performed by radial access (10 facilities) and those with ≤50% radial access cases (8 facilities). There was no difference in the rate of AKI between the groups (*p* = 0.11) using the propensity scores in the weighted analysis.

One of the variables used in our logistic regression model was hypotension derived from the first blood pressure record measured in the procedure room, but this variable was only collected starting with Version 5.0 of the CathPCI Registry. Because of this, 6,735 records from Version 4.0 were excluded from the analysis. To determine whether those missing hypotension records would have an impact on the overall analysis, we performed a second logistic regression analysis with new GBM propensity score weights without using the variable of hypotension. This increased the number of patient records in the analysis to 20,764 (RA = 11,637 and FA = 9,127), with the main result continuing to show that RA access was associated with a lower occurrence of AKI (OR 0.79 and 95% CI 0.73 to 0.86) and without significantly changing the other outcomes of the model (second model's C-statistic = 0.791). Accordingly, we focused our study on the results of the original model including hypotension.

## 4. Access Site and Predicted Risk of AKI

Comparison of the rates of AKI in the groups defined by the Mehran risk scores is shown in Table 2. Increasing risk of AKI is indicated by a higher numerical Mehran score. In all but the highest AKI risk group (Mehran score ≥16), RA access had a significantly lower incidence of AKI compared with FA access. In the highest risk group, RA access was numerically but not statistically lower (*p* = 0.10). However, compared with the other groups, the sample size in the highest risk group was considerably smaller which may partially explain the lack of significance.

## 5. Discussion

The main finding of our analysis was a significantly lower incidence of AKI after PCI when using RA access compared with FA access. The logistic regression model using the GBM propensity weighted variables showed an overall 18% reduction in the incidence of AKI with RA access. The only other variable associated with a lower incidence of AKI was male gender, but gender cannot be controlled by the operator. There was no association between the occurrence of AKI and a facility's experience with radial access. Tokarek and colleagues showed that operators performing a high percentage of RA access procedures had higher complications (death, stroke, and bleeding) with FA access procedures, but the occurrence of AKI was not evaluated in their study [25].

Analysis of data from the NCDR CathPCI Registry in prior studies identified several variables associated with a higher risk of AKI including the presence of cardiogenic shock, heart failure, diabetes, anemia, cardiac arrest before the procedure, PCI status, use of an intra-aortic balloon pump, and preprocedure creatinine, but these NCDR studies did not include the access site as a variable in the analysis [7, 26]. Our analysis identified these same variables as associated with AKI, thereby indirectly confirming the validity of our alternative statistical method using a GBM for propensity matching of the groups with RA or FA access.

Our finding of a decreased occurrence of AKI using RA access is congruent with some retrospective and meta-analyses examining the effect of radial access on AKI [9–11, 27, 28]. However, a lower incidence of AKI with RA access has not been a consistent finding among studies [13]. To date, there have been 2 randomized trials that evaluated the association of RA versus FA access on AKI in patients undergoing PCI, both in the setting of acute coronary syndromes [12, 14]. The AKI-MATRIX trial was a prespecified substudy of the randomized MATRIX trial and showed that RA access had a lower incidence of AKI defined as an absolute (>0.5 mg/dl) or a relative (>25%) increase in serum creatinine [12]. However, when applying the Kidney Disease: Improving Global Outcomes (KDIGO) definition, AKI remained less prevalent in the RA access patients, but the difference was not significant. AKI SAFARI was a post hoc analysis of data from the randomized SAFARI-STEMI trial and did not show a difference in AKI between RA and FA access using the KDIGO definition of AKI [14]. The occurrence of contrast-associated AKI was assessed in a substudy of the ADAPT-DES (Assessment of Dual AntiPlatelet Therapy With Drug Eluting Stents) study [29]. Contrary to other studies, RA access was found to be associated with development of AKI, but this result is tempered by a small number of patients in this study who had RA access. Because of these mixed results, the effect of RA access on the occurrence of AKI after PCI remains inconclusive.

The amount of radiographic contrast administered especially in patients with impaired renal function affects the occurrence of AKI [8]. Previously, it was shown that a simple ratio of contrast volume administered/creatinine clearance ≥3 substantially increased the likelihood of developing of AKI [30, 31]. Our analysis confirmed that both higher levels of preprocedure creatinine (OR 1.47) and an administered contrast volume/eGFR ≥3 (OR 1.47) were both associated with an increased occurrence of AKI.

Although we found an association between the diagnosis of NSTEMI and AKI following PCI (OR 1.28), we did not find an association with the diagnosis of STEMI. An association between the presence of a STEMI and AKI has not been a consistent finding among other studies [9, 11, 13, 14]. A STEMI presentation covers a wide range of acuity (from uncomplicated cases involving more distal branches of a vessel to proximal occlusion of a large artery with cardiogenic shock), and thus, variable results might be expected. Individual high-risk variables occurring with some STEMIs (such as cardiac arrest, shock, and pressor/mechanical support) may be better predictors of AKI than the presence of STEMI alone.

To evaluate if the benefit of RA access exists as the predicted risk of AKI increases, we determined the risk of AKI using the original Mehran risk score [20]. In all but the highest AKI risk group (Mehran scores ≥16), RA access had a significantly lower incidence of AKI compared with FA access. Even in the lowest risk group (Mehran score ≤5%), the occurrence of AKI was 1% lower with RA access compared with FA access and the benefit of RA access increased incrementally as the risk of AKI increased. In the highest risk group, RA access was roughly 9% lower with RA access, but the difference was not statistically lower (*p* = 0.10). However, compared with the other groups, the sample size in the highest risk group was considerably smaller which may partially explain the lack of significance. Mehran and colleagues did not consider the access site in this study as it was published in 2004 before RA access was widely used. Accordingly, their predicted risk of AKI likely reflects predominantly FA access procedures. It is noteworthy, however, that the predicted risk of AKI determined by the Mehran scores is consistently higher than the rate of AKI with FA access found in our study. Many factors could contribute to the lower current rate of AKI including use of less contrast material with the smaller size catheters now used, a better understanding of contrast volume limits, and better knowledge of ways to mitigate AKI. Mehran and colleagues have recently published an updated risk score based on 8 clinical variables [32]. The arterial access site was considered in their model but was later excluded by their use of stepwise selection. Moreover, their risk score was developed using their facility's internal database and contained some variables not captured in the NCDR registry and thus are not available for the development of our model. Accordingly, we used the original Mehran scores as a simple way to estimate the risk of AKI in our study cohort and demonstrating that the association of RA access and a lower rate of AKI exists across a spectrum of baseline renal function.

Although our study was not designed to determine the mechanism of the effect of RA access on AKI, three possibilities exist. Several studies have shown that RA access is associated with a reduction in postprocedure bleeding and the interaction between blood loss and the development of contrast-induced AKI has been examined [10, 15, 33]. Ohno and colleagues showed that postprocedure bleeding was significantly associated with contrast-induced AKI in patients undergoing PCI with the incidence of AKI increasing with bleeding severity [33]. In contrast, 2 other studies minimized the interaction of bleeding and contrast-induced AKI [10, 34]. Postprocedure bleeding was associated with AKI in our study (OR 3.94). The nearly 4-fold increase may occur because the development of bleeding following PCI potentially combines several factors contributing to AKI including hypotension, shock, anemia, and need for vasopressor support. Second, increasing amounts of contrast are associated with an increasing likelihood of developing AKI especially in those with impaired renal function [8]. It has been suggested that overall larger amounts of contrast are used with FA access, but this was not found in a meta-analysis of randomized trials comparing contrast use between RA and FA access [35]. Before adjustment, our FA group had approximately 15 ml more contrast used, but after adjustment, this was reduced to approximately 6 ml, an amount unlikely to have a clinical effect. Finally, an increased risk of cholesterol embolization to the kidneys occurring with catheter manipulation in the descending aorta during FA access has been suggested, but in a comparative study, no increase in cholesterol embolization was noted using FA access and determining the exact source of emboli is often difficult [36, 37].

## 6. Study Limitations

First, although the study cohort was derived from a large database using standardized NCDR definitions, it is a retrospective study. As with all retrospective database studies, the analysis is vulnerable to coding errors and missing values, the latter commonly noted in other retrospective studies [9–11, 13]. The ideal study would be a large, randomized trial of RA versus FA access, but given the established findings of less bleeding and lower mortality with RA access, it would be difficult to justify ignoring these advantages to develop a randomized study cohort [15–17]. Second, the timing of postprocedure creatinine blood samples and the amount, duration, and type of hydration used after the PCI were not standardized. These could affect the detection and occurrence of AKI, but these limitations likely occurred to a similar extent in both the RA and FA access groups. Finally, our study does not define the mechanism by which radial access lowers the risk of AKI.

Although not randomized, there are advantages to our study. The inclusion of all indications for PCI rather than just acute coronary syndromes and the large sample size allow for greater generalization of our findings. To compensate for the lack of randomization, we used a novel but a well-established machine learning method, GBM, to generate propensity score weights and reduce selection bias. GBM provides a more robust model to generate propensity scores than simple matching of patient characteristics because it utilizes multiple decision trees, and each tree focuses on reducing the errors of the previous trees [21–23]. In addition, utilizing the GBM to develop propensity score weights allowed our analysis to retain all of the study participants without limiting the size of the groups by one-to-one propensity score matching.

## 7. Conclusions

Our analysis showed that RA access is associated with a lower incidence of AKI following PCI by roughly 18% compared with FA access. This was shown using a method of propensity score weighting not previously used and supports existing literature showing the advantages of RA access on the development of AKI. Moreover, the advantage of RA access on the development of AKI exists over a wide predefined range of risk levels of AKI. These data support the use of RA access as opposed to FA access when technically possible to reduce the occurrence of AKI.

## Figures and Tables

**Table 1:** Odds ratios from logistic regression on AKI after PCI with propensity score weights.

Variables	Odds ratio	Wald 95% CI	
Postprocedure bleeding event	3.94	3.13	4.97
Presence of shock	2.82	2.24	3.57
Presence of heart failure	2.14	1.93	2.38
Presence of cardiac arrest	2.08	1.59	2.70
Use of pharmacologic vasopressor support	2.03	1.63	2.53
Higher PCI status (elective, urgent, emergency, and salvage)	1.78	1.55	2.05
Diabetes	1.69	1.52	1.88
IABP	1.63	1.24	2.14
Anemia	1.58	1.41	1.77
Preprocedure creatinine	1.47	1.37	1.59
Contrast volume/eGFR ≥3	1.47	1.31	1.64
**Other vascular complications**	**1.42**	**0.82**	**2.46**
NSTEMI	1.28	1.11	1.48
Black	1.19	1.03	1.37
**STEMI**	**1.15**	**0.91**	**1.45**
**Prior cerebrovascular disease**	**1.06**	**0.93**	**1.21**
**PCI multivessel disease**	**1.05**	**0.95**	**1.16**
Age	1.02	1.02	1.03
**BMI**	**1.01**	**1.00**	**1.01**
**Hypotension (first recorded BP in procedure room)**	**0.94**	**0.67**	**1.32**
Arterial access (radial)	0.82	0.74	0.91
Gender (male)	0.80	0.72	0.89

Rows highlighted in bold were considered not significant. Abbreviations: BMI = body mass index, BP = blood pressure, CI = confidence interval, eGFR = estimated glomerular filtration rate, IABP = intra-aortic balloon pump, NSTEMI = non-ST-segment elevation myocardial infarction, STEMI = ST-segment elevation myocardial infarction, and PCI = percutaneous coronary intervention.

**Table 2:** AKI by the access site and Mehran risk group.

*N*	Mehran score (predicted risk of AKI)	Arterial access site	AKI rate (%)	*p*
8,053	≤5	Radial	2.7	0.02
	7.5%^*∗*^	Femoral	3.7	

3,994	6–10	Radial	7.3	0.03
	14.0%^*∗*^	Femoral	9.2	

1,676	11–15	Radial	14.1	<0.01
	26.1%^*∗*^	Femoral	20.3	

354	≥16	Radial	29.4	0.10
	57.3%^*∗*^	Femoral	38.3	

^*∗*^The percentages shown are the risk of developing AKI calculated from the Mehran risk score as reported in the original manuscript [20]. Abbreviations: AKI = acute kidney injury.

## Data Availability

The data used to support the findings of this study are available from the corresponding author upon request.

## Supplementary Materials

Supplementary Materials Supplementary Table 1: explanation of how the fields shown were recorded to streamline the analysis portion of the study. Supplementary Table 2: standardized mean difference for all variables before and after weighting.
