# Supplementary material for: Association of Radial Artery Access with Reduced Incidence of Acute Kidney Injury
Source: J Interv Cardiol. 2023 Jan 18;2023:1117379. doi: 10.1155/2023/1117379 (PMC9876675; doi:10.1155/2023/1117379)
Supplement: Supplementary Materials — Supplementary Table 1: explanation of how the fields shown were recorded to streamline the analysis portion of the study. Supplementary Table 2: standardized mean difference for all variables before and after weighting. [file 1117379.f1.zip › Supplementary Table 1.docx]

**Supplementary Table 1**

**Data Translation Stage**

The following fields were recoded to streamline the analysis portion of the study.

| Race | Three groups were used: 1 - Caucasian/White (%) and 2 - African American/Black (%). All other race entries were categorized as 3 - Other (%). |
| --- | --- |
| IABP | Is a combined field. Yes included records that had IABP marked yes or had IABP listed under Mechanical Ventricular Support Devices. All other records were marked as No for IABP. |
| Cardiac Arrest | Is a combined field. Yes included records that had Cardiac Arrest within 24 Hours marked Yes or Cardiac Arrest Witnessed marked Yes. All other records were marked as No for Cardiac Arrest |
| Shock | Recoded from Cardio Shock Status. Yes included records with Transient or Sustained shock. All other records were marked No for Shock. |
| Bleeding Event | Recoded the Any Bleeding Event within 72 Hours. Yes included records marked Yes for any bleeding event within 72 hours. All other records were marked as No for Bleeding Event. |
| CABG | Yes included records that indicated a CABG event happened on the same admission. All other records were recorded as No for CABG. Those identified as Yes for this field were excluded from the analysis |
| Arterial Access | Femoral and Radial arterial access were the only two options included in the analysis. There were 51 records with another type of access recorded and these were excluded from the analysis |
| Pre-procedure hypotension | Recoded from systolic BP. Yes included records with a Systolic BP < 90 mmHg recorded as the first BP in the procedure room. Since the systolic BP field contained over 6700 blank records, we did not include them in the analysis. All records with a Systolic BP > 90 mmHg were recorded as No for hypotension. |
| Heart Failure | Yes included records that indicated a Heart Failure event. No included records marked No or records that were blank. We acknowledge the limitation of this assumption, but feel that if a Heart Failure had occurred it would have been marked Yes and the blanks could reasonably be assumed to be No. |
| PCI Multivessel Disease | Is a combined field. If PCI Multivessel Disease was marked as Yes and the Multivessel Procedure Type was marked as Initial PCI, then the record was marked as Yes for PCI Multivessel Disease. Records in the Multivessel Procedure Type marked as Staged PCI were excluded from the analysis. All other records were marked as no for PCI Multivessel Disease. |
| Contrast Volume 3xGFR | Recoded field from Contrast Volume. Yes included records with a Contrast Volume > 3 times the eGFR. |
| Arterial Cross Over | Yes included records that indicated an arterial cross over. Records that were blank for this field were recoded as No for Arterial Cross Over. We acknowledge the limitation of this assumption, but feel that if a cross over event had occurred it would have been marked Yes and the blanks could reasonably be assumed to be No. Records marked as Yes for Arterial Cross Over were excluded. |

Abbreviations: BP = Blood Pressure, CABG = Coronary Artery Bypass Surgery, eGFR = Estimated Glomerular Filtration Rate, IABP = Intraaortic Balloon Pump, PCI = Percutaneous Coronary Intervention
