# Supplementary material for: Association of Radial Artery Access with Reduced Incidence of Acute Kidney Injury
Source: J Interv Cardiol. 2023 Jan 18;2023:1117379. doi: 10.1155/2023/1117379 (PMC9876675; doi:10.1155/2023/1117379)
Supplement: Supplementary Materials — Supplementary Table 1: explanation of how the fields shown were recorded to streamline the analysis portion of the study. Supplementary Table 2: standardized mean difference for all variables before and after weighting. [file 1117379.f1.zip › Supplementary Table 2.docx]

**Supplementary Table 2:
Standardized mean difference for all variables before and after weighting**

| **Clinical Variables** | **Standardized Mean Difference (SMD)**  **Before Weighting** | **SMD  After Weighting** |
| --- | --- | --- |
| Age (years) | 27.5% | 0.7% |
| eGFR (ml/min/1.73m^2^) | -9.2% | 1.6% |
| BMI (kg/m^2^) | -11.3% | -0.2% |
| Pre-procedure creatinine (mg/dL) | 11.8% | -1.4% |
| Heart failure | 31.3% | 2.9% |
| Anemia | 25.5% | 1.5% |
| Diabetes | 14.3% | 1.4% |
| Prior cerebrovascular disease | 22.9% | 2.0% |
| Contrast Volume/eGFR ≥ 3 | 22.5% | 7.0% |
| Shock | 16.7% | 0.0% |
| Other vascular complications | 16.7% | 0.0% |
| Gender | -15.2% | 0.0% |
| Race | 8.6% | 2.0% |
| PCI Status | -18.2% | -4.3% |
| IABP | 9.1% | 6.3% |
| Hypotension | 10.0% | 0.0% |
| Bleeding Event | 15.4% | 5.6% |
| Cardiac Arrest | 7.7% | 5.3% |
| NSTEMI | 2.0% | -1.4% |
| STEMI | -16.3% | -3.3% |
| PCI Multivessel | 16.0% | 7.1% |
| Vasopressor Support | 15.8% | 7.4% |

Standardized mean difference was calculated for all variables to display the effectiveness of balancing the arterial access groups using the generalized bosted model propensity score weights. Binary variables which were entered as 1 for yes and 0 for no appear in the weighted columns as a numbers between 0 and 1. All variables were analyzed in the final logistic regression appropriately based on their numeric or categorial nature. A positive difference indicates a higher Femoral Access value.

Abbreviations: BMI = body mass index, eGFR = estimated glomerular filtration rate, IABP = Intraaortic balloon pump, NSTEMI = non-ST-segment elevation myocardial infarction, SMD = standardized mean difference, STEMI = ST-segment elevation myocardial infarction, PCI = percutaneous coronary intervention
